# Identification of candidate genes for leaf scorch in *Populus deltoids* by the whole genome resequencing analysis

**DOI:** 10.1038/s41598-018-33739-7

**Published:** 2018-11-06

**Authors:** Weibing Zhuang, Tianyu Liu, Shenchun Qu, Binhua Cai, Yalong Qin, Fengjiao Zhang, Zhong Wang

**Affiliations:** 1 Jiangsu Key Laboratory for the Research and Utilization of Plant Resources, Institute of Botany, Jiangsu Province and Chinese Academy of Sciences, Nanjing, 210014 China; 2 College of Horticulture, Nanjing Agricultural University, Nanjing, 210095 China

## Abstract

Leaf scorch exists as a common phenomenon in the development of plant, especially when plants encounter various adversities, which leads to great losses in agricultural production. Both Jinhong poplar (JHP) and Caihong poplar (CHP) (*Populus deltoids*) are obtained from a bud sport on Zhonghong poplar. Compared with CHP, JHP always exhibits leaf scorch, poor growth, premature leaf discoloration, and even death. In this study, the candidate genes associated with leaf scorch between JHP and CHP were identified by the whole genome resequencing using Illumina HiSeqTM. There were 218,880 polymorphic SNPs and 46,933 indels between JHP and CHP, respectively. Among these, the candidate genes carrying non-synonymous SNPs in coding regions were classified into 6 groups. The expression pattern of these candidate genes was also explored in JHP and CHP among different sampling stages. Combined with the qRT-PCR analysis, the results showed that genes associated with transport of various nutritional elements, senescence and MYB transcription factor might play important roles during the process of leaf scorch in *Populus deltoids*. Four genes belonging to these three groups carried more than three SNPs in their coding sequence, which might play important roles in leaf scorch. The above results provided candidate genes involved in leaf scorch in *Populus deltoids*, and made us better understand the molecular regulation mechanism of leaf scorch in *Populus deltoids*.

## Introduction

Leaf scorch exists as a common phenomenon in the development of plant, especially when plants encounter various adversities, which leads to great losses in agricultural production. In summer, Ginkgo and Maple often exhibits leaf scorch, poor growth, premature leaf discoloration, and some of them often die. Many crops, such as cotton, soybeans and corn, also have leaf scorch phenomenon, especially in summer, which also leads to great losses in agricultural production. Many factors can lead to leaf scorch in many kinds of crops and trees, such as high temperature, drought, pests, diseases and so on. Therefore, it is of great significance to explore the mechanism of leaf scorch in plants and to avoid it. However, the molecular mechanism of leaf scorch in crops and trees are still unclear and need us to be further explored.

The Populus plants, naturally distributed in the Northern Hemisphere, are important economic species. In addition to reforestation, they can also provide raw materials for pulp, biofuel and paper industries^1^. Since the first color-leaf poplar (Zhonghong poplar) was cultivated in 2006, several kinds of colorful leaf poplar cultivars were cultivated, which has a wide range of applications in the courtyard embellishment, road greening, garden set King and so on^2^. Among them, two *Populus deltoids* cultivars, Jinhong poplar (JHP) and Caihong poplar (CHP), obtained from a bud sport of the same color-leaf poplar (Zhonghong poplar), attracted researchers’ attention. JHP and CHP had a similar leaf color at early stage of growth, and significant differences occurred between them at the late stage of growth. Compared with CHP, JHP always exhibits leaf scorch, premature leaf discoloration, poor growth, and even death within one or two years. As these two color-leaf poplars had similar genetic background, studying the genome differences of these two color-leaf poplars is a better way to explore the candidate genes associated with leaf scorch in poplar. In addition, the genome of *Populus trichocarpa* has been released, which can provide more reference when we explore the molecular regulation mechanism occurred in *Populus deltoids*^3^. Therefore, JHP and CHP are good materials to revel the molecular mechanism of leaf scorch in poplar.

As the development of next-generation sequencing technologies, plant omics sequencing has become common platforms to explore various biological processes in poplar, such as wood development, a variety of biotic or abiotic stress, reproductive development and so on^4,5^. At present, more than 900 Populus genotypes have been successfully resequenced, which make us discover large scale SNPs and polymorphisms^6^. However, up to now, there is no report on the molecular mechanism of leaf scorch by the whole genome resequencing, and the molecular mechanism of leaf scorch in *Populus deltoids* is still obscure.

In present study, we explored the molecular regulation mechanism of leaf scorch between JHP and CHP with the whole genome resequencing. Many SNPs and indels between them are discovered, and the candidate genes carrying non-synonymous SNPs in coding regions were identified combined with the expression patterns of genes carrying non-synonymous SNPs in coding regions. The results could provide us deeper insight into the molecular regulation mechanism of leaf scorch in *Populus deltoids*, and could reduce the loss caused by the leaf scorch in agricultural production in future.

## Results

### Screening of genome variation between JHP and CHP

The genome variation between JHP and CHP was explored through an Illumina Genome Analyzer. There were 104.1 million reads (about 15.6 Gb) for JHP compared with the reference genome, which can give 36.7 × sequencing depth. For the CHP, there was 108.9 million reads (16.3 Gb) compared with the reference genome, which can reach 38.4 × sequencing depth. 410.6 Mb consensus sequence were produced from the 99.2 million reads (accounting for 95.2% of total reads) in the JHP genome, and 410.8 Mb consensus sequence were produced from the 103.5 million reads (accounting for 95.0% of total reads) in the CHP genome. Both JHP and CHP had the same genome coverage compared with the reference genome, which was 95.8% (Table 1).

**Table 1:** The screening of genome variation between JHP and CHP compared with the reference genome based on the whole genome resequencing.

	JHP	CHP
Total reads	104,104,539	108,920,101
Total size (bp)	4,659,991,276	4,876,051,559
Sequencing depth	36.7261×	38.4297×
Mapped reads	99,154,015 (95.2%)	103,464,008 (95.0%)
Properly paired reads	97,083,229	101,402,196
Consensus sequence (bp)	410,633,296	410,845,308
Genome coverage	95.8%	95.8%

### The summary of SNPs between JHP and CHP on individual chromosomes compared with the reference genome

There were many SNPs between JHP and CHP on individual chromosomes compared with the reference genome, and 218,880 polymorphic SNPs between JHP and CHP was discovered. The frequencies of SNPs between JHP and CHP on individual chromosomes are much different. Chromosome 09 had the fewest SNPs (4940), while chromosome 01 had the most SNPs (23,443) (Table 2).

**Table 2:** Summary of SNPs between JHP and CHP on individual chromosomes compared with the reference genome.

Chr. No.	No. of polymorphic SNPs	No. of intergenic regions	Genic region					No. of genes
			Total	UTR	CDS		No. of introns	
					Synonymous	Non- synonymous		
1	23443	20799	2644	245	360	573	1466	2492
2	13346	10727	2619	399	309	395	1516	1455
3	10093	8443	1650	154	231	417	848	1197
4	11596	10225	1371	159	199	330	683	1263
5	10495	9319	1176	148	168	287	573	1315
6	9905	8485	1420	135	179	309	797	1401
7	7142	6256	886	92	140	202	452	791
8	7852	6892	960	128	128	187	517	1048
9	4940	3831	1109	186	159	190	574	793
10	8761	7602	1159	144	151	215	649	1161
11	9780	8449	1331	94	241	414	582	1020
12	9862	8324	1538	184	198	282	874	831
13	6810	5882	928	111	104	188	525	824
14	8794	7643	1151	110	152	279	610	944
15	10622	8173	2449	319	363	430	1337	920
16	6967	6119	848	109	134	235	367	759
17	10044	8479	1565	134	287	509	635	959
18	7793	6796	997	107	145	290	555	866
19	10388	8918	1470	122	235	370	743	850
Scaffolds	30247	27136	3111	218	540	1006	1347	1399
Total	218880	188498	30382	3298	4423	7108	15650	22288

Among 218,880 polymorphic SNPs, there were 188,498 polymorphic SNPs in intergenic regions, accounting for 85.5% of total polymorphic SNPs, and 30,382 polymorphic SNPs in genic regions, accounting for 14.5% of total polymorphic SNPs (Table 2). In genic regions of 30,382 polymorphic SNPs, intron regions, untranslated regions (UTRs) and coding sequence (CDS) regions accounted for 15,650, 3298 and 11,531 polymorphic SNPs, respectively. There was 38.4% synonymous SNPs and 61.6% non-synonymous SNPs in 11,531 polymorphic SNPs of CDS regions. The functions of genes encoding amino acids were changed due to the missense mutation or nonsense mutation, and 22,288 genes may be affected by the polymorphic SNPs between JHP and CHP (Table 2).

The detected homozygote polymorphic SNPs were analyzed based on the nucleotide substitutions. Two kinds of nucleotide substitutions occurred including transversions (A/C, A/T, C/G, and G/T) and transitions (A/G and C/T). In 11045 homozygote polymorphic SNPs, there was 7895 transitions, accounting for 71.5%, and 3150 transversions, accounting for 28.5%, which indicated that transitions occurred much easily than transversions. For the transversions, A/T had a highest frequency (9.6%), and C/G had a lowest frequency (3.7%) (Table 3).

**Table 3:** The types and ratio of nucleotide substitutions in detected homozygote polymorphic SNPs between JHP and CHP.

	SNPs	Ratio (%)
**Transitions**		
A/G	3906	35.4
C/T	3989	36.1
**Transversions**		
A/C	822	7.4
A/T	1065	9.6
C/G	407	3.7
G/T	856	7.8
Total	11045	100

### The summary of indels between JHP and CHP on individual chromosomes compared with the reference genome

46,933 polymorphic indels occurred in poplar chromosomes between JHP and CHP compared with the reference genome, and the number of polymorphic indels on each chromosome varied from 1428 (chromosome 16) to 4846 (chromosome 01) (Table 4). In accordance with the largest number of SNPs (23,443) on chromosome 01, there also was the greatest number of polymorphic indels on it (Tables 2 and 4). 37,840 of 46,933 polymorphic indels occurred in intergenic regions, accounting for 80.1%, while 9093 of them took place in genic regions, accounting for 19.9%. For 9093 polymorphic indels in genic regions, the region of intron had the largest number of polymorphic indels with 6631, UTRs followed with 1752, and CDS region had the smallest number of polymorphic indels with 710 (Table 4). The results showed that most polymorphic indels in the total polymorphic indels were located in intergenic regions, and most polymorphic indels in the genic regions were located in intron regions. 16,976 genes may be affected by the polymorphic indels between JHP and CHP due to the frameshift mutation.

**Table 4:** Summary of indels between JHP and CHP on individual chromosomes compared with the reference genome.

Chr. No.	No. of polymorphic indels	No. of intergenic regions	Genic region				No. of genes
			Total	UTR	CDS	No. of introns	
1	4846	3944	902	157	61	684	1843
2	3386	2637	749	151	49	549	1176
3	2413	1897	516	104	46	366	935
4	2409	1954	455	84	34	337	962
5	2457	1981	476	107	44	325	997
6	2475	1922	553	95	37	421	1114
7	1578	1294	284	37	20	227	610
8	1851	1382	469	94	28	347	842
9	1516	1126	390	96	35	259	650
10	2114	1635	479	79	36	364	931
11	1917	1594	323	69	22	232	714
12	2222	1737	485	90	39	356	689
13	1528	1207	321	62	18	241	622
14	1871	1471	400	67	29	304	725
15	2582	1952	630	129	50	451	730
16	1428	1170	258	73	16	169	538
17	1820	1496	324	77	25	222	675
18	1772	1417	355	71	30	254	677
19	2015	1712	303	47	28	228	624
Scaffolds	4733	4312	421	63	63	295	922
Total	46933	37840	9093	1752	710	6631	16976

**Figure 1:**
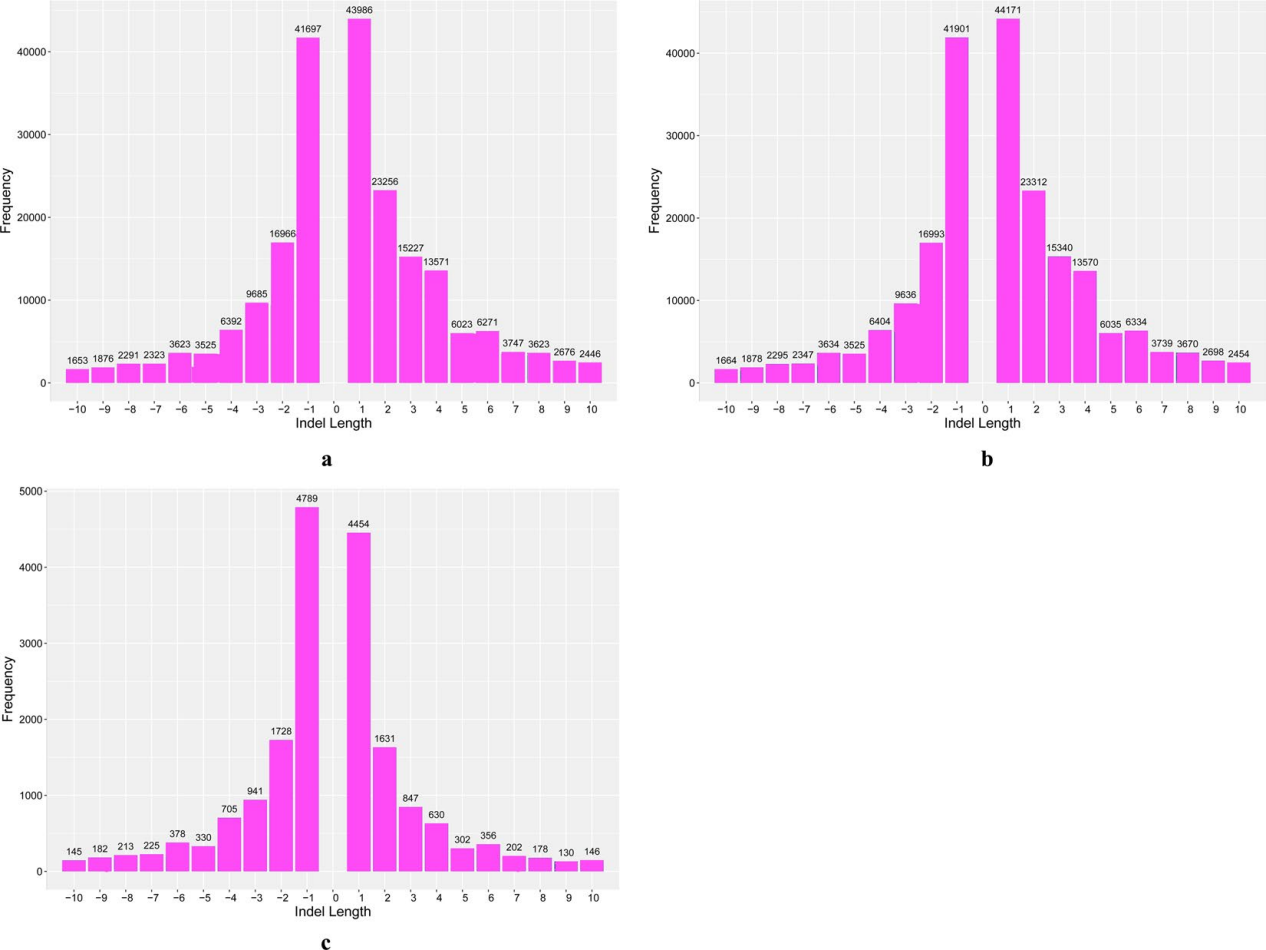
The lengths and corresponding frequency of indels among reference genome sequence of poplar, JHP and CHP. (**a**) Shows the lengths and corresponding frequency of indels between reference genome sequence of poplar and CHP genome sequence; (**b**) shows the lengths and corresponding frequency of indels between reference genome sequence of poplar and JHP genome sequence; (**c**) shows the lengths and corresponding frequency of indels between JHP and CHP genome sequence.

The lengths and corresponding frequency of deletions and insertions (1–10 bp) among reference genome sequence of poplar, JHP and CHP were calculated (Fig. 1). There was 211027 indels between reference genome sequence of poplar and CHP genome sequence, and mononucleotide, dinucleotide, trinucleotide and tetranucleotide indels accounted for 40.6%, 19.1%, 11.8% and 9.5%, respectively (Fig. 1a). There was 211600 indels between reference genome sequence of poplar and JHP genome sequence, and mononucleotide, dinucleotide, trinucleotide and tetranucleotide indels accounted for 40.7%, 19.0%, 11.8% and 9.4%, respectively (Fig. 1b). The frequency and length of homozygote polymorphic indels between JHP and CHP were also evaluated (Fig. 1c). There was less difference between the frequency of deletions and insertions, while a larger difference between the frequency of indels and the corresponding lengths of indels. Mononucleotide indels between JHP and CHP accounting for 49.9% had the most frequent indels. The frequency of dinucleotide indels, trinucleotide indels and tetranucleotide indels accounted for 18.1%, 9.7% and 7.2%, respectively. The results showed that there was a negative relationship between the lengths of indels and the corresponding frequency of indels (Fig. 1).

### Gene ontology analysis of genes carrying polymorphic SNPs and indels

**Figure 2:**
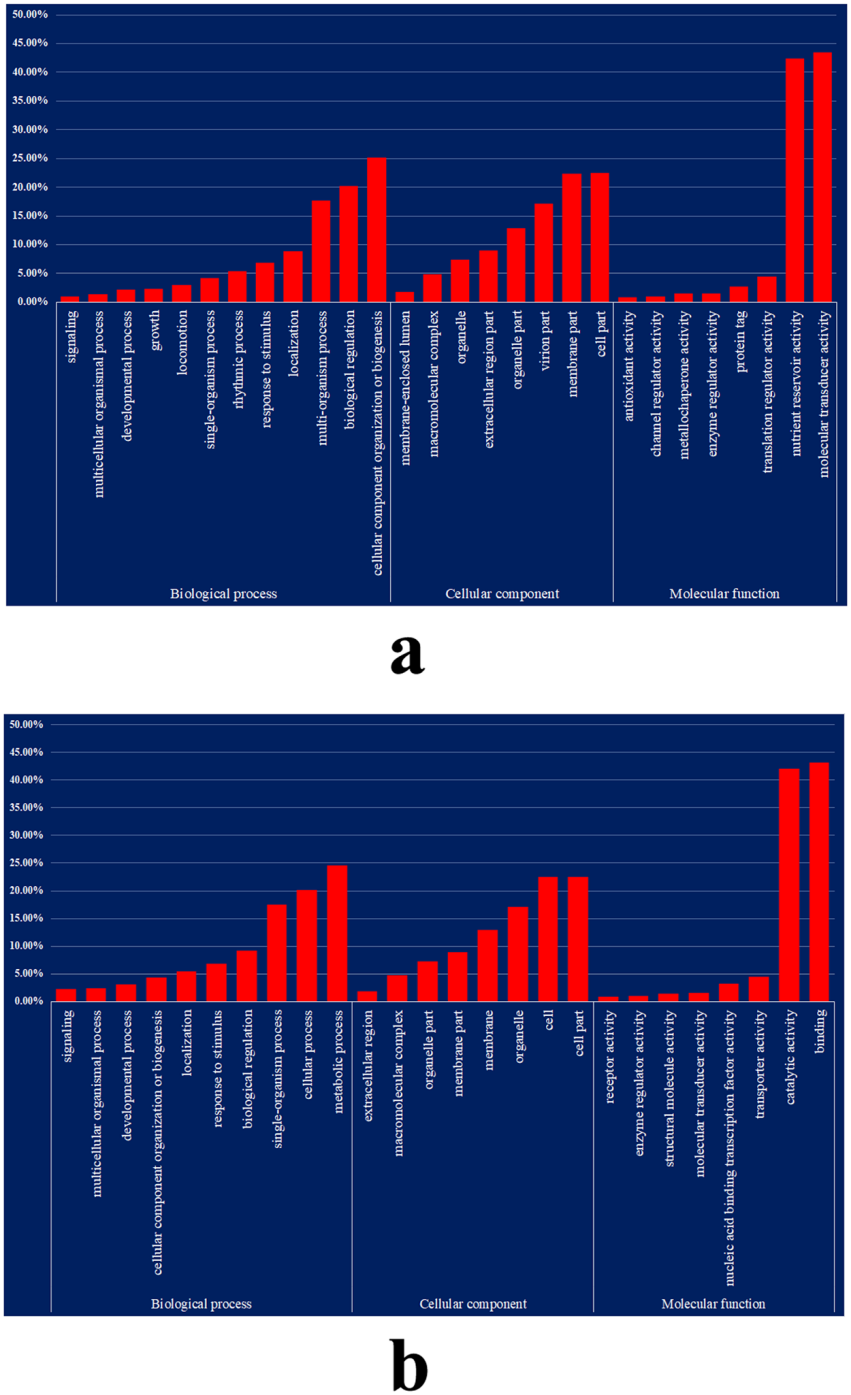
Gene ontology (GO) functional enrichment analysis of SNPs or indels between JHP and CHP. (**a**) Shows the GO term representation (%) of SNPs; (**b**) shows the GO term representation (%) of indels.

The genes carrying polymorphic SNPs or indels between JHP and CHP were categorized into three functional groups based on gene ontology: molecular function, cellular component, and biological process (Fig. 2). For the genes carrying polymorphic SNPs, the significant enrichment categories according to molecular function were binding (7821), catalytic activity (7638), transporter activity (807), nucleic acid binding transcription factor activity (483), molecular transducer activity (261) and structural molecule activity (167) (Fig. 2a). These genes were also classified according to cellular components as follows: cell part (5258), cell (5240), organelle (3996), membrane (3011), membrane part (2097), organelle part (1713), macromolecular complex (1139), and extracellular region (423). The major categories of biological processes were metabolic process (9159), cellular process (7371), single-organism process (6438), biological regulation (3236), response to stimulus (2507), localization (1973), cellular component organization or biogenesis (1545), developmental process (1076), multicellular organismal process (835), signaling (785), and reproductive process (499), respectively (Fig. 2a).

For the genes carrying polymorphic indels, the significant enrichment categories on the basis of molecular function were binding (6003), catalytic activity (5852), transporter activity (626), nucleic acid binding transcription factor activity (448), molecular transducer activity (226), structural molecule activity (190), and enzyme regulator activity (133) (Fig. 2b). The genes were classified according to cellular components as follows: cell part (4167), cell (4155), organelle (3164), membrane (2389), membrane part (1650), organelle part (1338), macromolecular complex (887), and extracellular region (326). On the basis of biological processes, metabolic process (7057), cellular process (5789), single-organism process (5038), biological regulation (2629), response to stimulus (1964), localization (1576), cellular component organization or biogenesis (1262), developmental process (889), multicellular organismal process (684), and signaling (638) were the major categories.

### Screening of genes related to leaf scorch in *Populus deltoids*

**Figure 3:**
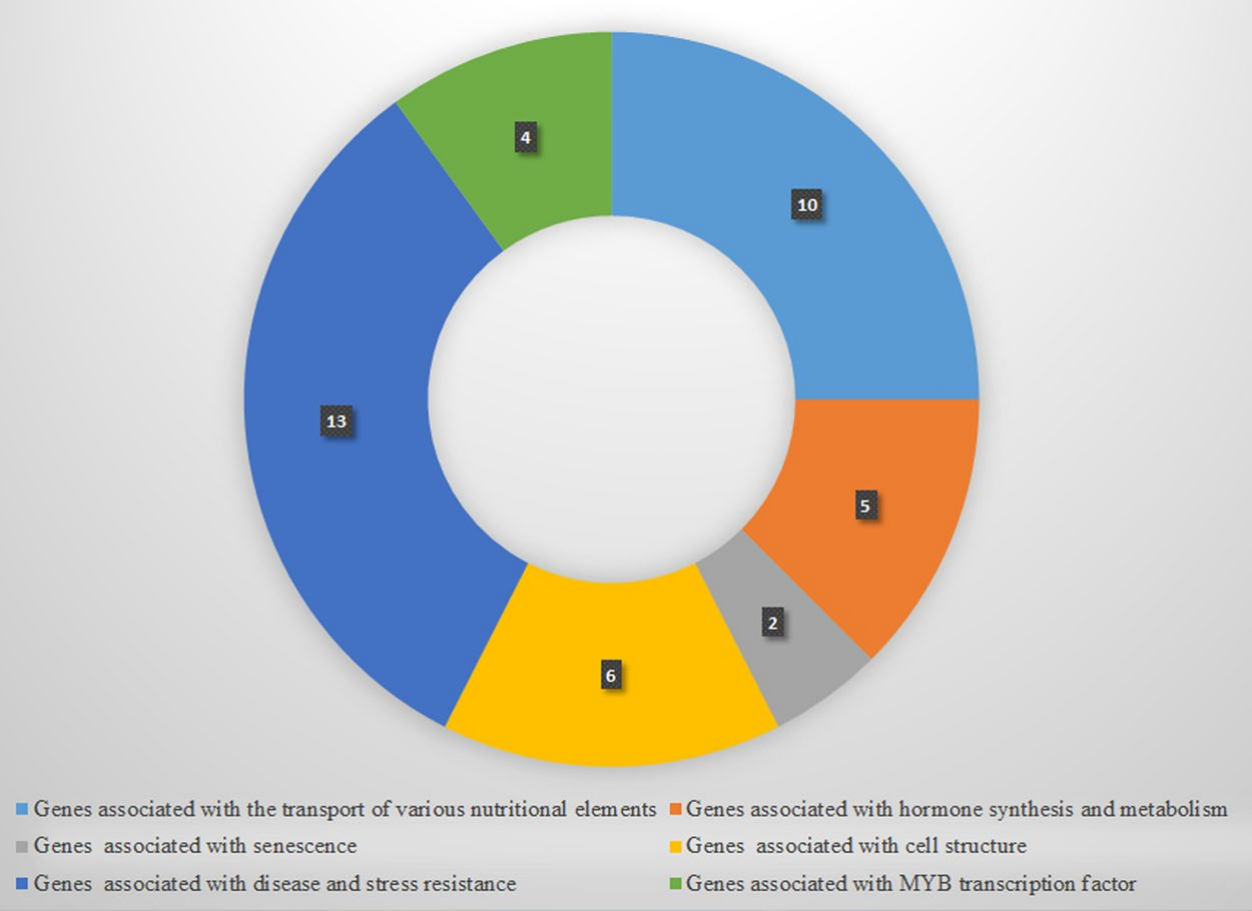
Functional categorization of candidate genes carrying non-synonymous SNPs in coding regions. The numbers in the figure indicates the number of genes in each subgroup.

Many genes are affected due to the polymorphic SNPs or indels between JHP and CHP. As many reasons can lead to the leaf scorch, such as high temperature, drought, pests, diseases and so on, we screened 40 affected genes carrying non-synonymous SNPs in coding regions, which might be associated with leaf scorch, and divided them into six groups: genes associated with the transport of various nutritional elements (10), genes associated with hormone synthesis and metabolism (5), genes associated with senescence (2), genes associated with cell structure (6), genes associated with disease and stress resistance (13), and genes associated with MYB transcription factor (4) (Fig. 3).

### The expression levels of candidate genes which carried non-synonymous SNPs in coding regions on different sampling date in JHP and CHP

**Figure 4:**
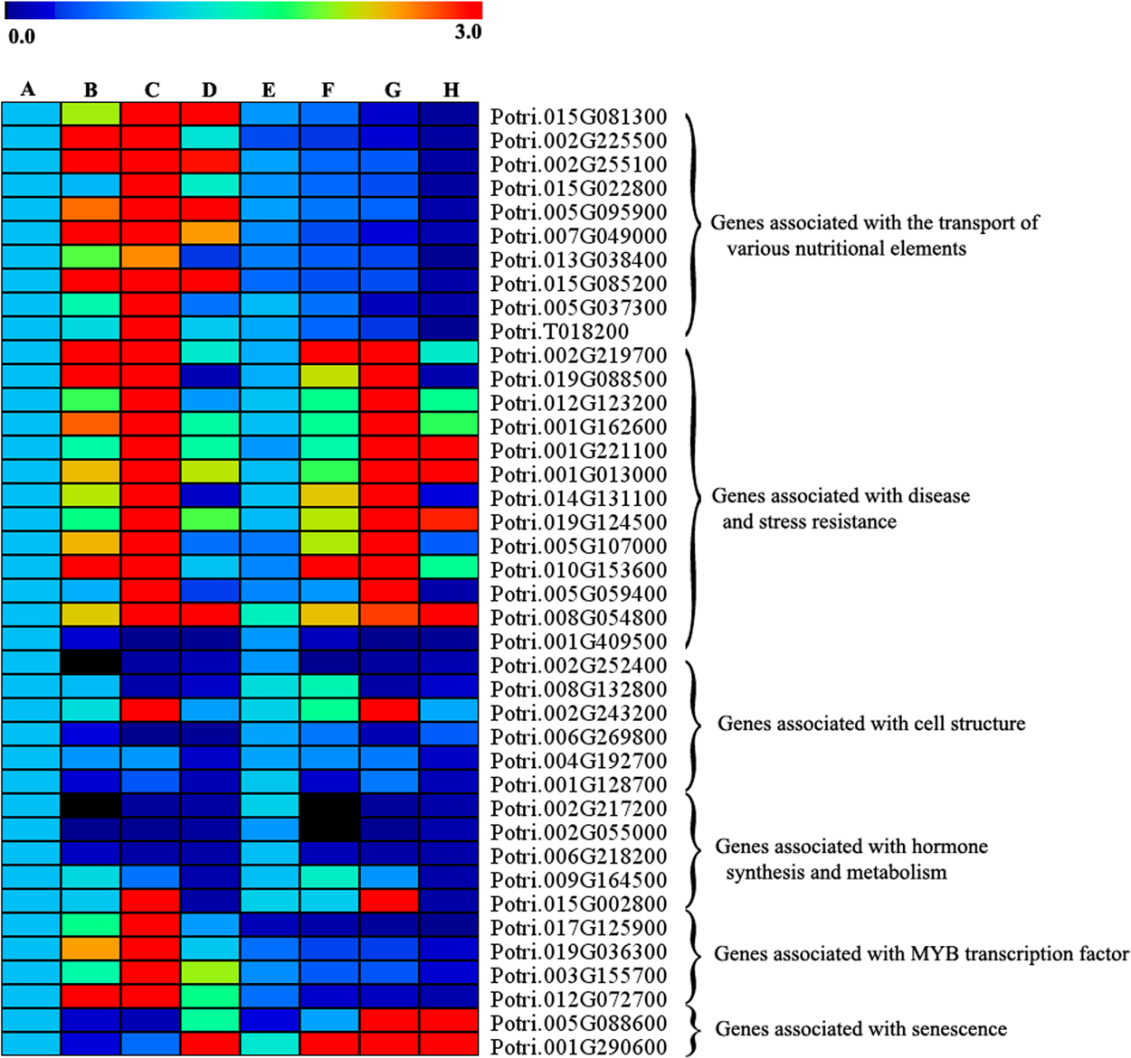
The heatmap of expression levels for the candidate genes associated with leaf scorch in poplar. The relative expression value of genes was calculated with 2^−ΔΔCt^ method, and presented from low relative expression value (blue) to high relative expression value (red) on a color scale (0.0 to 3.0). The intensity of the colors is proportional to the gene relative expression value. Each row represents the expression level of gene, and each column represents the samples collecting date. (**A**–**D**) Represents the samples of CHP collected on 5 June, 5 July, 5 August and 5 September, 2016 respectively, and (**E**–**H**) represents the samples of JHP collected on 5 June, 5 July, 5 August and 5 September, 2016 respectively.

The qRT-PCR was performed to reveal the expression difference of candidate genes which might be involved in the process of leaf scorch. The expression level of most genes associated with the transport of various nutritional elements, in CHP gradually increased form 5 June to 5 August, 2016, and decreased form 5 August to 5 September, 2016 (Fig. 4, Table S1). While the expression level of those genes in JHP decreased form 5 June to 5 September, 2016, and the expression level of those genes in JHP was much lower than those of genes in CHP. The expression of most genes associated with MYB transcription factor had a similar trend with the expression of most genes associated with the transport of various nutritional elements (Fig. 4). Genes associated with senescence had different trends compared with the expression of most genes associated with the transport of various nutritional elements and MYB transcription factor (Fig. 4). The expression levels of most genes associated with senescence increased gradually from 5 June to 5 August, 2016, and increased sharply from 5 August to 5 September, 2016 in JHP, and the expression of most genes associated with senescence had a slight fluctuation form 5 June to 5 August, 2016 in CHP, and increased gradually from 5 August to 5 September, 2016 (Fig. 4). Different from that most genes associated with the transport of various nutritional elements, senescence and MYB transcription factor had a completely different expression levels on different sampling date in JHP and CHP, genes associated with hormone synthesis and metabolism, cell structure and disease and stress resistance had a similar expression levels on different sampling date in JHP and CHP (Fig. 4). Therefore, genes associated with the transport of various nutritional elements, senescence and MYB transcription factor could play important roles in the process of leaf scorch in poplar. The non-synonymous SNPs in coding regions of these genes were also summarized (Table S2), and high affinity nitrate transporter 2.5 had four SNPs in coding region between JHP and CHP. Compared with the genes in CHP, there are substitutions from G to C, C to A, G to C and G to A in JHP, which lead to the substitutions from Gly to Ala, Leu to Ile, Gly to Ala and Val to Ile, respectively. There are three SNPs in coding region of the protein NRT1/PTR FAMILY 6.4 between JHP and CHP, which has substitutions from T to C, G to A, and C to A in JHP, which lead to the substitutions from Phe to Leu, Gly to Ser and Ser to Cys. For manganese-dependent ADP-ribose/CDP-alcohol diphosphatase, there are also three SNPs in coding region between JHP and CHP, which has substitutions from T to A, G to A, and T to A in JHP, leading to the substitutions from Asp to Glu, Cys to Tyr and Ile to Asn. There are four SNPs in coding region of senescence-associated carboxylesterase 101 (Precursor) between JHP and CHP, and the substitutions from C to T, G to A, T to A and T to A in JHP, which lead to the substitutions from His to Tyr, Ser to Asn, Asn to Lys and Cys to Ser, respectively (Table S2). For the other genes associated with the transport of various nutritional elements, senescence and MYB transcription factor just have one SNPs in coding region between JHP and CHP.

## Discussion

Leaf scorch is a common phenomenon in agricultural production, especially when plants suffer from a variety of biotic or abiotic stress. In our results, many genes associated with the transport of various nutritional elements carried non-synonymous SNPs in coding regions, which might change the proteins’ functions of those genes or their gene expression, and those genes might play important roles in plant growth and development, especially in leaf senescence.

Nitrogen (N) is an important mineral nutrient, which plays crucial roles in plant growth and development^7^. NRT2.5, a high-affinity NO_3_^−^ transporter, is highly expressed in shoots after pathogen infection and during senescence (www.genevestigator.com). NRT2.1 and NRT2.6 were also reported to take some roles during the process of pathogen infection^8,9^. In our results, the expression level of NRT2.5 increased from 5 June to 5 August, and decreased from 5 August to 5 September, 2016 in CHP, while decreased form 5 June to 5 September, 2016 in JHP. The expression level of NRT2.5 in CHP was much higher than that of JHP, which might be the reason why JHP had a leaf scorch phenomenon (Fig. 4). NRT1/PTR FAMILY genes were originally identified as nitrate or di/tri-peptide transporters, and now they were proved to be that they can carry plant hormones and some secondary metabolites, such as abscisic acid (ABA), gibberellin (GA), auxin (indole-3-acetic acid), glucosinolates, and so on^10^. NRT1/PTR FAMILY 6.4, identified in our results, carried non-synonymous SNPs in coding regions. The affected NRT1/PTR FAMILY 6.4 gene might have some effects on the transportation of plant hormones, and then lead to the leaf scorch in JHP. *AMT1–*3, a high-affinity NH4^+^ transporter gene, was induced under low nitrogen in rice roots, and was repressed with nitrogen supplementation^11,12^. The rice overexpressing *OsAMT1.3* had longer roots with a higher area, volume, and number of tips, which might promote the transgenic rice to better adapt to a variety of biotic or abiotic stress^13^. In our research, several genes associated with the nitrogen transporters were identified. The expression level of those genes in CHP was much higher than that of JHP, which indicated that the lower expression level of those genes in JHP may disturb their normal growth, and exhibit the leaf scorch.

Potassium (K), another important mineral nutrient, play pivotal roles on both vegetative growth and reproductive growth in plants^14^. As K transport across the plasma membranes and vacuolar takes directly roles on the turgor regulation, and cell elongation is driven by turgor pressure, therefore, K translocators is crucial for growth^15^. In our study, probable potassium transporter 13 carrying non-synonymous SNPs in coding regions between JHP and CHP, were also identified. Compared with the higher expression level of probable potassium transporter 13 in CHP, the expression level of probable potassium transporter 13 in JHP was also lower, which might retard the establishment of tip growth, leading to its leaf scorch.

Cadmium/zinc-transporting ATPase HMA1, a member of P(IB)-ATPase family, is located on the chloroplast envelope. As they can take roles on the transport of copper, zinc, cadmium and cobalt, they are essential for plant growth and development processes^16–18^. In our study, two genes carrying non-synonymous SNPs in coding regions associated with plant transport of cadmium and zinc were identified. As the transport of cadmium was pivotal for the growth of plant, those two genes were also important candidate genes associated with leaf scorch in JHP.

In our results, two genes associated with sugar transporter, which carried non-synonymous SNPs in coding regions, were identified between JHP and CHP. Similar with the other genes associated with the transport of various nutritional elements, those two genes also had a lower expression level in JHP compared with those in CHP (Fig. 4). Sugar transporter family ERD6-like homologs (At1g08920 and At1g08930) could be involved in anthocyanin pigmentation in a Ca^2+^ signal-dependent manner^19^. Chang *et al*., (2007) found that the dark-treated plants, with no photosynthetic assimilates, have to alter their carbohydrate chemistry and this may require an increased expression of the sugar-transporter gene^20^. Therefore, those two genes might be pivotal candidate genes associated with leaf scorch in JHP.

MYB genes constitute one of the largest families of transcription factors in plants and regulate many aspects of plant biology, such as primary and secondary metabolism, cell fate, developmental processes and responses to biotic and abiotic stresses^21,22^. Four genes associated with MYB transcription factor were identified in our study, which carried non-synonymous SNPs in coding regions. The expression pattern of those genes in JHP and CHP were very similar, however, the four MYB genes had a higher expression levels compared with those in JHP. WEREWOLF and GLABRA1, belonging to MYB transcription factors, are involved in root hair cell differentiation and determining cell fate during trichome, respectively^23,24^. AtMYB77 regulates lateral root formation, and ASYMMETRIC LEAVES1 regulates leaf patterning and shoot morphogenesis^25,26^. Recently, several R2R3 MYB transcription factors have been found to regulate secondary cell wall biosynthesis in *Populus spp*. (poplar) and *Eucalyptus spp*. (Eucalyptus)^27–29^. Arabidopsis overexpression AtMYB88 can promote their tolerance to abiotic stress by restricting the divisions that occur late in the stomatal cell lineage^30^. As MYB genes play important roles in many aspects of plant biology, genes associated with MYB transcription factor might be candidate genes for leaf scorch in JHP.

Leaf senescence, controlled by environmental and intrinsic factors, leads to a sequence of events such as chlorophyll loss, dismantling of cellular, degradation of macromolecules and cell death^31–33^. Cysteine proteases (CPs), the most abundant class of proteases, were consistently up-regulated during natural or induced senescence in different plant species^34,35^. There was an increase of cysteine proteases activities in senescing leaves of Arabidopsis, wheat, cowpea and soybean^36–38^. And in the senescing wheat leaves, more than four vacuolar CPs were induced at continuous darkness^37^. There was an increase of PLCPs (papain-like cysteine protease) gene expression in senescing soybean, sweet potato and barley leaves^39–42^. In addition, transcript and protein levels of PLCP SAG12 are induced in the senescing leaves with a limitation of N^43^. In addition, the suppression of BoCP5, a close homologue of the senescence-associated cysteine protease, can delay their floret senescence^44^. In our results, senescence-specific cysteine protease and senescence-associated carboxylesterase 101 genes had much higher expression levels in JHP than those in CHP, and they have a different expression patterns in JHP and CHP. These two genes in JHP were expressed increased slightly from 5 June to 5 August, 2016, and increased sharply from 5 August to 5 September, 2016. However, these two genes in CHP maintained a relative stable level from 5 August to 5 September, and increased slightly from 5 August to 5 September, 2016. As those two genes play important roles in the senescing tissues, which might be pivotal candidate genes associated with leaf scorch in JHP.

In addition, there was much higher frequency of non-synonymous substitutions compared with that of synonymous substitutions for the polymorphic SNPs in CDS regions, which is in accordance with previous results^45,46^. The functions of candidate genes carrying non-synonymous SNPs in CDS regions might change due to the changes of amino acid sequences, which could change the phenotype of plant. Therefore, the genes carrying non-synonymous SNPs in CDS regions in our results might be involved in the leaf scorch in poplar.

## Materials and Methods

### Plant materials

**Figure 5:**
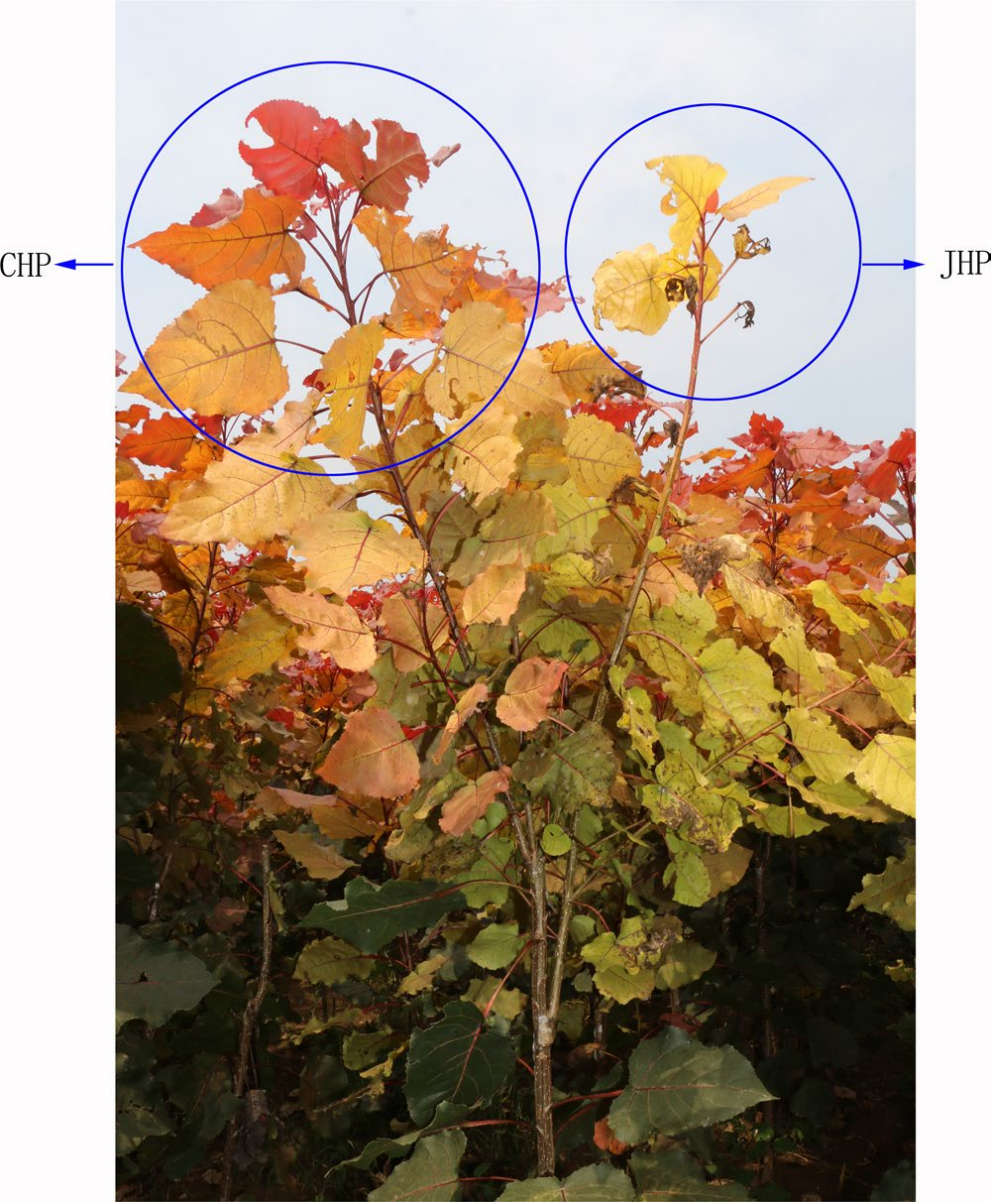
The phenotype of JHP and CHP (grafted on the Zhonghong poplar) on 5 September, 2016. The branch in the left circle belongs to the CHP, and the branch in the right circle belongs to the JHP.

Two *Populus deltoids* cultivars, JHP and CHP, resulting from a bud sport of Zhonghong poplar, had similar leaf color. However, compared with CHP, JHP always exhibits leaf scorch, premature leaf discoloration, and they usually die within one or two years. We collected the leaves of JHP and CHP respectively on 5 June, 2016, and the DNA was extracted to perform the whole genome resequencing. To better understanding the regulation of leaf scorch in *Populus deltoids*, we grafted JHP and CHP onto the same rootstock (Zhonghong poplar). The leaves of JHP showed leaf scorch, premature leaf discoloration, and most of them fallen on 5 September, 2016, while leaves of CHP had a normal growth state, and fallen on 5 November, 2016 (Fig. 5). The leaves of JHP and CHP were collected respectively on 5 June, 5 July, 5 August and 5 September, 2016, and the RNA was extracted to perform the qRT-PCR to evaluate the expression levels of genes associated with leaf scorch in *Populus deltoids*.

### DNA library construction and massively parallel sequencing

Genomic DNA was extracted from the fresh leaves of JHP and CHP with the modified method from Kim^47^. The analysis of whole genome resequencing was conducted according the method modified from Lee^45^. The target DNA fragments (400–500 bp) were randomly sheared from the genomic DNA by Covaris S2 (Covaris, Woburn, MA, USA), and were evaluated using a Bioanalyzer 2100 (Agilent Technologies, USA). After fragmentation, the blunt ends were obtained from the resulting overhangs, and were cleaned with AMPure XP beads (Beckman Coulter Genomics, Danvers, MA, USA). In order to lower self-ligation among blunt fragments and enhance the ligation ratios between index adapters and fragmented DNA, the 3′ ends were adenylated, and immediately ligated to the index adapters. The target products were obtained after gel extraction and purification. To further improve the target fragments, PCR were conducted with the specific adapter primers. To obtain the target fragments (500–600 bp), the fragments were cleaned again and evaluated with AMPure XP beads and Agilent Bioanalyzer 2100. The target fragments were sequenced on the Hiseq4000 platform (Illumina, CA, USA), and the base calling and data analysis were conducted according to the Illumina pipeline with default settings.

### Preprocessing

After massively parallel sequencing, the obtained sequences were demultiplexed and trimmed with Solexa QA package v.1.13^48^. To get better results of bases, two things are needed to be considered. Firstly, the index adapter sequences should be removed. Secondly, low quality reads should be discarded when one of the paired-end read had more than 0.05 probability of error, or when the Phred quality score had less than Q = 20.

### Alignment and analysis of variants including SNPs

The obtained reads were aligned to the released poplar genome with the Burrows–Wheeler Aligner (BWA) program^49^, which can be downloaded from https://phytozome.jgi.doe.gov/pz/portal.html. The genome of *Populus trichocarpa* is divided into 19 chromosomes, which is approximately 422.9 Mb. There are 42,950 loci containing protein-coding transcripts and 63,498 protein-coding transcripts in the genome of *Populus trichocarpa*. The parameters of BWA program are set as following: the gap extension penalty, gap open penalty, mismatch penalty, number of threads, maximum differences in the seed and seed length were 8, 15, 6, 10, 1, and 32, respectively, and the other parameters of BWA program are set as default values. SAMtools were used to acquire high quality BWA-mapped reads from the resulting BAM file, and the single nucleotide polymorphism (SNP) are called from these reads^50^. The parameters of varFilter command to call SNPs which had variable positions were as following: the minimum SNP quality, minimal mapping quality, the maximum read depths and the minimum read depths were 100, 30, 1000 and 5, respectively. The significant sites were acquired from the called SNP positions with a custom perl script.

### RNA extraction and qRT-PCR analysis of genes associated with leaf scorch

Total RNA was extracted from fresh leaves of JHP and CHP (100 mg) with the modified method from Pavy^51^. The concentration of extracted RNA was evaluated by BioPhotometer (Eppendorf) at the absorbance of 260 nm. The ratio of A260/A280 and A260/A230 for a good purity of RNA ranged from 1.80 to 2.05, and from 2.00 to 2.60, respectively, and the integrity of RNA was determined by the agarose gel electrophoresis. The obtained RNA was stored at −70 °C until further use.

The expression levels of candidate genes associated with leaf scorch was evaluated through qRT-PCR. According to the manufacturer’s instructions, a sample of total RNA (1 μg) was reverse transcribed for first-strand cDNA synthesis using a ReverTra Ace qPCR RT Kit (Toyobo). Gene-specific primers were designed using Primer Premier 5.0 software according to the sequence of the target gene in the *Populus* genome database (Table S3). The qRT-PCR was conducted on the Applied Biosystems 7300, and the volume of reaction was 20 μl, including 10 μl of SYBRR Premix Ex Taq™ (Perfect Real Time; TaKaRa), 0.3 μl (10 pM) of each primer, 1 μl of 10-fold-diluted cDNA and 8.4 μl of sterile double-distilled water. The program for the qRT-PCR were as following: 95 °C for 3 min, 95 °C for 25 s with 35 cycles, 62 °C for 25 s, and 72 °C for 40 s. The *UBQ* gene was used as control gene to normalize gene expression^52^. The relative expression level of genes was analyzed with the 2^−ΔΔCt^ method, which represented the difference of the cycle threshold (Ct) between the control UBQ products and target gene products. Data analyses were performed with the software of SPSS 17.0, and three biological replicates were conducted for each set of conditions in JHP and CHP.

## Conclusions

In present study, the whole genome resequencing was performed to explore the candidate genes associated with leaf scorch between JHP and CHP with Illumina HiSeqTM 4000. 218,880 polymorphic SNPs and 46,933 indels were identified between JHP and CHP, respectively. The candidate genes associated with leaf scorch between JHP and CHP, carrying non-synonymous SNPs in coding regions, were classified into 6 groups. Combined with the qRT-PCR results, genes associated with the transport of various nutritional elements (10), MYB transcription factor (4) and senescence (2) were further identified. Four genes belonging to these three groups carried more than three SNPs in their coding sequence, which might play important roles in leaf scorch. The above results provided candidate genes involved in leaf scorch in *Populus deltoids*, and could provide some directions to explore the molecular mechanism of leaf scorch in poplar.

## Electronic supplementary material

Table S1

Table S2

Table S3
