# Supplementary material for: Identification of candidate genes for leaf scorch in Populus deltoids by the whole genome resequencing analysis
Source: Sci Rep. 2018 Nov 6;8:16416. doi: 10.1038/s41598-018-33739-7 (PMC6219557; doi:10.1038/s41598-018-33739-7)
Supplement: Supplementary file 1 — Table S1 [file 41598_2018_33739_MOESM1_ESM.docx]

**Identification of candidate genes for leaf scorch in *Populus deltoids* by the whole genome resequencing analysis**

**Weibing Zhuang^1 *^, Tianyu Liu^2^, Shenchun Qu^2^, Binhua Cai^2^, Yalong Qin^1^, Fengjiao Zhang^1^, Zhong Wang^1^**

**^1^** Jiangsu Key Laboratory for the Research and Utilization of Plant Resources, Institute of Botany, Jiangsu Province and Chinese Academy of Sciences, Nanjing 210014, China

**^2^** College of Horticulture, Nanjing Agricultural University, Nanjing 210095, China

*Corresponding author

Telephone numbers: +86-25-84397131

Fax numbers: +86-25-84395266

E-mail address: [wanmeiabc@hotmail.com](mailto:wanmeiabc@hotmail.com)

**Table S1**. The relative expression value of candidate genes associated with leaf scorch presented in Figure 4. A to D represents the samples of CHP collected on 5 June, 5 July, 5 August and 5 September, 2016 respectively, and E to H represents the samples of JHP collected on 5 June, 5 July, 5 August and 5 September, 2016 respectively.

| **Gene name** | **Accession** | **A** | **B** | **C** | **D** | **E** | **F** | **G** | **H** |
| --- | --- | --- | --- | --- | --- | --- | --- | --- | --- |
| **Genes associated with the transport of various nutritional elements** | | | | | | | | | |
| High affinity nitrate transporter 2.5 | Potri.015G081300 | 1 | 2.119 | 7.345 | 3.302 | 0.783 | 0.568 | 0.221 | 0.05 |
| Protein NRT1/ PTR FAMILY 6.4 | Potri.002G225500 | 1 | 3.918 | 14.59 | 1.266 | 0.418 | 0.338 | 0.248 | 0.059 |
| Ammonium transporter 1 member 2 | Potri.002G255100 | 1 | 8.535 | 25.58 | 2.895 | 0.839 | 0.548 | 0.487 | 0.072 |
| Inorganic phosphate transporter 1-11 | Potri.015G022800 | 1 | 0.959 | 12.24 | 1.338 | 0.754 | 0.551 | 0.435 | 0.059 |
| Probable potassium transporter 13 | Potri.005G095900 | 1 | 2.639 | 29.18 | 4.093 | 0.824 | 0.614 | 0.532 | 0.105 |
| Probable cadmium/zinc-transporting ATPase HMA1, chloroplastic (Precursor) | Potri.007G049000 | 1 | 5.952 | 21.41 | 2.485 | 0.717 | 0.419 | 0.274 | 0.12 |
| Calcium-transporting ATPase 12, plasma membrane-type | Potri.013G038400 | 1 | 1.919 | 2.543 | 0.34 | 0.654 | 0.495 | 0.397 | 0.032 |
| Manganese-dependent ADP-ribose/CDP-alcohol diphosphatase | Potri.015G085200 | 1 | 4.746 | 39.4 | 13.58 | 0.564 | 0.446 | 0.425 | 0.1 |
| Sugar transporter ERD6-like 2 | Potri.005G037300 | 1 | 1.474 | 3.333 | 0.609 | 0.966 | 0.589 | 0.155 | 0.085 |
| Sugar carrier protein C | Potri.T018200 | 1 | 1.178 | 6.869 | 1.067 | 0.877 | 0.536 | 0.333 | 0.028 |
| **Genes associated with disease and stress resistance** | | | | | | | | | |
| GDSL esterase/lipase At2g38180 (Precursor) | Potri.002G219700 | 1 | 3.936 | 4.511 | 1.313 | 0.91 | 3.732 | 6.32 | 1.329 |
| Laccase-14 (Precursor) | Potri.019G088500 | 1 | 3.498 | 32.9 | 0.14 | 0.916 | 2.214 | 18.98 | 0.113 |
| Putative disease resistance protein RGA3 | Potri.012G123200 | 1 | 1.853 | 4.297 | 0.792 | 1.019 | 1.625 | 5.005 | 1.613 |
| Pectinesterase 1 (Precursor) | Potri.001G162600 | 1 | 2.682 | 14.09 | 1.519 | 1.002 | 1.591 | 10.56 | 1.828 |
| Thaumatin-like protein 1 (Precursor) | Potri.001G221100 | 1 | 1.512 | 16.91 | 1.54 | 0.783 | 1.485 | 12.5 | 3.109 |
| Peroxidase C3 (Precursor) | Potri.001G013000 | 1 | 2.384 | 27.03 | 2.178 | 0.991 | 1.845 | 18.77 | 3.038 |
| Stress-related protein | Potri.014G131100 | 1 | 2.168 | 15.45 | 0.211 | 1.009 | 2.329 | 12.1 | 0.302 |
| Wound-responsive protein GWIN3 (Precursor) | Potri.019G124500 | 1 | 1.662 | 19.65 | 1.896 | 0.979 | 2.173 | 16.6 | 2.848 |
| Mechanosensitive ion channel protein 2, chloroplastic (Precursor) | Potri.005G107000 | 1 | 2.412 | 3.741 | 0.596 | 0.629 | 2.163 | 3.846 | 0.495 |
| Pleiotropic drug resistance protein 3 | Potri.010G153600 | 1 | 3.34 | 16.49 | 1.021 | 0.705 | 3.053 | 9.826 | 1.595 |
| Chitinase 2 | Potri.005G059400 | 1 | 0.929 | 7.945 | 0.359 | 0.694 | 0.785 | 6.821 | 0.1 |
| Heat shock cognate 70 kDa protein 2 | Potri.008G054800 | 1 | 2.308 | 3.698 | 5.515 | 1.395 | 2.362 | 2.77 | 3.371 |
| Chitin-inducible gibberellin-responsive protein 1 | Potri.001G409500 | 1 | 0.254 | 0.013 | 0.03 | 0.797 | 0.162 | 0.017 | 0.04 |
| **Genes associated with cell structure** | | | | | | | | | |
| Vegetative cell wall protein gp1 (Precursor) | Potri.002G252400 | 1 | 0.01 | 0.077 | 0.135 | 0.779 | 0.012 | 0.069 | 0.127 |
| Cell number regulator 2 | Potri.008G132800 | 1 | 0.979 | 0.1 | 0.209 | 1.203 | 1.454 | 0.087 | 0.214 |
| Extensin (Precursor) | Potri.002G243200 | 1 | 1.206 | 8.897 | 0.818 | 1.12 | 1.591 | 5.528 | 0.877 |
| 65-kDa microtubule-associated protein 3 | Potri.006G269800 | 1 | 0.302 | 0.018 | 0.04 | 0.849 | 0.62 | 0.141 | 0.505 |
| Wall-associated receptor kinase-like 8 (Precursor) | Potri.004G192700 | 1 | 0.77 | 0.786 | 0.209 | 0.855 | 0.737 | 0.645 | 0.192 |
| Cell division control protein 48 homolog A | Potri.001G128700 | 1 | 0.252 | 0.469 | 0.136 | 1.05 | 0.216 | 0.63 | 0.152 |
| **Genes** **associated with hormone synthesis and metabolism** | | | | | | | | | |
| Auxilin-related protein 2 | Potri.002G217200 | 1 | 0.011 | 0.058 | 0.082 | 1.115 | 0.009 | 0.051 | 0.102 |
| Auxin response factor 6 | Potri.002G055000 | 1 | 0.014 | 0.033 | 0.059 | 0.785 | 0.011 | 0.03 | 0.113 |
| Ethylene-responsive transcription factor ERF017 | Potri.006G218200 | 1 | 0.185 | 0.091 | 0.092 | 0.998 | 0.152 | 0.084 | 0.098 |
| ABSCISIC ACID-INSENSITIVE 5-like protein 1 | Potri.009G164500 | 1 | 1.17 | 0.607 | 0.116 | 1 | 1.347 | 0.774 | 0.105 |
| Gibberellin 20 oxidase 2 | Potri.015G002800 | 1 | 1.055 | 8 | 0.09 | 1.117 | 1.079 | 7.568 | 0.092 |
| **Genes associated with MYB transcription factor** | | | | | | | | | |
| Transcription factor MYB114 | Potri.017G125900 | 1 | 1.64 | 4.846 | 0.803 | 0.149 | 0.102 | 0.051 | 0.02 |
| Transcription repressor MYB5 | Potri.019G036300 | 1 | 2.474 | 6.438 | 1.05 | 0.577 | 0.384 | 0.358 | 0.217 |
| Transcription factor MYB86 | Potri.003G155700 | 1 | 1.505 | 7.551 | 2.104 | 0.739 | 0.489 | 0.47 | 0.241 |
| Transcription factor bHLH79 | Potri.012G072700 | 1 | 4.337 | 30.2 | 1.659 | 0.58 | 0.219 | 0.185 | 0.106 |
| **Genes associated with senescence** |  |  |  |  |  |  |  |  |  |
| Senescence-specific cysteine protease SAG39 | Potri.005G088600 | 1 | 0.221 | 0.13 | 1.562 | 0.304 | 0.849 | 3.638 | 57.68 |
| Senescence-associated carboxylesterase 101 (Precursor) | Potri.001G290600 | 1 | 0.287 | 0.565 | 3.927 | 1.289 | 2.976 | 20.73 | 32 |
