# Supplementary material for: Identification of candidate genes for leaf scorch in Populus deltoids by the whole genome resequencing analysis
Source: Sci Rep. 2018 Nov 6;8:16416. doi: 10.1038/s41598-018-33739-7 (PMC6219557; doi:10.1038/s41598-018-33739-7)
Supplement: Supplementary file 2 — Table S2 [file 41598_2018_33739_MOESM2_ESM.docx]

**Identification of candidate genes for leaf scorch in *Populus deltoids* by the whole genome resequencing analysis**

**Weibing Zhuang^1 *^, Tianyu Liu^2^, Shenchun Qu^2^, Binhua Cai^2^, Yalong Qin^1^, Fengjiao Zhang^1^, Zhong Wang^1^**

**^1^** Jiangsu Key Laboratory for the Research and Utilization of Plant Resources, Institute of Botany, Jiangsu Province and Chinese Academy of Sciences, Nanjing 210014, China

**^2^** College of Horticulture, Nanjing Agricultural University, Nanjing 210095, China

*Corresponding author

Telephone numbers: +86-25-84397131

Fax numbers: +86-25-84395266

E-mail address: [wanmeiabc@hotmail.com](mailto:wanmeiabc@hotmail.com)

**Table S2**. Identification of functional mutations in coding regions of poplar genes which are associated with leaf scorch.

| **Gene name** | **Accession** | **SNP position** | **Ref**. | **CHP/JHP** | **Non-syn type** |
| --- | --- | --- | --- | --- | --- |
| **Genes associated with the transport of various nutritional elements** | | | | | |
| High affinity nitrate transporter 2.5 | Potri.015G081300 | 10572343 | C | C/G | Ala/Gly |
|  |  | 10573269 | A | A/C | Ile/Leu |
|  |  | 10573285 | G | C/G | Ala/Gly |
|  |  | 10573326 | A | A/G | Ile/Val |
| Protein NRT1/ PTR FAMILY 6.4 | Potri.002G225500 | 21444959 | C | C/T | Leu/Phe |
|  |  | 21445363 | A | A/G | Ser/Gly |
|  |  | 21446049 | C | A/C | Cys/Ser |
| Ammonium transporter 1 member 2 | Potri.002G255100 | 24443450 | G | A/G | Met/Val |
| Inorganic phosphate transporter 1-11 | Potri.015G022800 | 1768893 | C | C/G | Ser/Cys |
| Probable potassium transporter 13 | Potri.005G095900 | 7212930 | T | A/T | Ile/Phe |
| Probable cadmium/zinc-transporting ATPase HMA1, chloroplastic (Precursor) | Potri.007G049000 | 4678228 | T | T/A | Tyr/Asn |
| Calcium-transporting ATPase 12, plasma membrane-type | Potri.013G038400 | 2625632 | G | A/G | Asp/Gly |
| Manganese-dependent ADP-ribose/CDP-alcohol diphosphatase | Potri.015G085200 | 10882314 | A | A/T | Glu/Asp |
|  |  | 10882528 | A | A/G | Tyr/Cys |
|  |  | 10882633 | T | A/T | Asn/Ile |
| Sugar transporter ERD6-like 2 | Potri.005G037300 | 2745549 | T | T/G | Val/Gly |
| Sugar carrier protein C | Potri.T018200 | 160216 | A | A/T | Arg/Trp |
| **Genes associated with disease and stress resistance** | | | | | |
| GDSL esterase/lipase At2g38180 (Precursor) | Potri.002G219700 | 20472047 | G | G/A | Glu/Lys |
| Laccase-14 (Precursor) | Potri.019G088500 | 12027689 | A | G/A | Val/Ile |
| Putative disease resistance protein RGA3 | Potri.012G123200 | 14242595 | T | G/T | Val/Leu |
|  |  | 14242610 | C | T/C | Trp/Arg |
|  |  | 14242785 | T | T/C | Phe/Ser |
|  |  | 14242791 | G | G/T | Arg/Ile |
| Pectinesterase 1 (Precursor) | Potri.001G162600 | 13596202 | T | A/T | Tyr/Phe |
|  |  | 13597775 | A | G/A | Ala/Thr |
|  |  | 13598062 | A | A/C | Glu/Asp |
| Thaumatin-like protein 1 (Precursor) | Potri.001G221100 | 22697698 | T | T/C | Ser/Pro |
| Peroxidase C3 (Precursor) | Potri.001G013000 | 883818 | A | A/G | Asn/Ser |
| Stress-related protein | Potri.014G131100 | 10004144 | A | A/C | His/Pro |
| Wound-responsive protein GWIN3 (Precursor) | Potri.019G124500 | 15236630 | A | A/T | Stop codon/Cys |
| Mechanosensitive ion channel protein 2, chloroplastic (Precursor) | Potri.005G107000 | 8193001 | C | A/C | Gln/Lys |
| Pleiotropic drug resistance protein 3 | Potri.010G153600 | 16121449 | C | C/A | Leu/Ile |
|  |  | 16125184 | G | G/C | Glu/Asp |
| Chitinase 2 | Potri.005G059400 | 4181971 | A | A/C | Tyr/Ser |
| Heat shock cognate 70 kDa protein 2 | Potri.008G054800 | 3233391 | A | A/T | Ser/Cys |
|  |  | 3235121 | T | A/T | Asn/Tyr |
| Chitin-inducible gibberellin-responsive protein 1 | Potri.001G409500 | 43310052 | G | T/G | Asp/Glu |
| **Genes associated with cell structure** | | | | | |
| Vegetative cell wall protein gp1 (Precursor) | Potri.002G252400 | 24164347 | G | G/C | Gly/Ala |
| Cell number regulator 2 | Potri.008G132800 | 8792905 | A | A/G | Asp/Ser |
| Extensin (Precursor) | Potri.002G243200 | 23547744 | G | A/G | Ile/Met |
| 65-kDa microtubule-associated protein 3 | Potri.006G269800 | 27172967 | G | A/G | Lys/Glu |
| Wall-associated receptor kinase-like 8 (Precursor) | Potri.004G192700 | 20719343 | G | C/G | Asn/Glu |
|  |  | 20719353 | C | G/C | Glu/Asp |
| Cell division control protein 48 homolog A | Potri.001G128700 | 10418091 | G | A/G | Ser/Gly |
| **Genes** **associated with hormone synthesis and metabolism** | | | | | |
| Auxilin-related protein 2 | Potri.002G217200 | 20051760 | G | T/G | Asp/Glu |
| Auxin response factor 6 | Potri.002G055000 | 3672320 | T | G/T | Gln/His |
| Ethylene-responsive transcription factor ERF017 | Potri.006G218200 | 23061092 | T | A/T | Glu/Asp |
|  |  | 23061097 | A | A/G | Asn/Ser |
| ABSCISIC ACID-INSENSITIVE 5-like protein 1 | Potri.009G164500 | 12595187 | T | T/A | Asp/Glu |
| Gibberellin 20 oxidase 2 | Potri.015G002800 | 190006 | C | A/C | Glu/Asp |
| **Genes associated with MYB transcription factor** | | | | | |
| Transcription factor MYB114 | Potri.017G125900 | 13807940 | A | A/G | Ile/Thr |
| Transcription repressor MYB5 | Potri.019G036300 | 4088254 | G | T/G | Ser/Arg |
| Transcription factor MYB86 | Potri.003G155700 | 16836808 | C | A/C | Asn/Thr |
| Transcription factor bHLH79 | Potri.012G072700 | 9823322 | A | G/A | Gly/Asp |
| **Genes associated with senescence** | | | | | |
| Senescence-specific cysteine protease SAG39 | Potri.005G088600 | 6626987 | A | T/A | Stop codon/Lys |
| Senescence-associated carboxylesterase 101 (Precursor) | Potri.001G290600 | 29621139 | C | T/C | Tyr/His |
|  |  | 29621209 | A | A/G | Asn/Ser |
|  |  | 29621216 | T | A/T | Lys/Asn |
|  |  | 29622478 | A | A/T | Ser/Cys |
