# Supplementary material for: Identification of candidate genes for leaf scorch in Populus deltoids by the whole genome resequencing analysis
Source: Sci Rep. 2018 Nov 6;8:16416. doi: 10.1038/s41598-018-33739-7 (PMC6219557; doi:10.1038/s41598-018-33739-7)
Supplement: Supplementary file 3 — Table S3 [file 41598_2018_33739_MOESM3_ESM.docx]

**Identification of candidate genes for leaf scorch in *Populus deltoids* by the whole genome resequencing analysis**

**Weibing Zhuang^1 *^, Tianyu Liu^2^, Shenchun Qu^2^, Binhua Cai^2^, Yalong Qin^1^, Fengjiao Zhang^1^, Zhong Wang^1^**

**^1^** Jiangsu Key Laboratory for the Research and Utilization of Plant Resources, Institute of Botany, Jiangsu Province and Chinese Academy of Sciences, Nanjing 210014, China

**^2^** College of Horticulture, Nanjing Agricultural University, Nanjing 210095, China

*Corresponding author

Telephone numbers: +86-25-84397131

Fax numbers: +86-25-84395266

E-mail address: [wanmeiabc@hotmail.com](mailto:wanmeiabc@hotmail.com)

**Table S3**. Specific primers used in relative quantitative real-time RT-PCR.

| **Gene name** | **Accession** | **Forward primer (5’to 3’)** | **Reverse primer (5’ to 3’)** |
| --- | --- | --- | --- |
| **Genes associated with the transport of various nutritional elements** | | | |
| High affinity nitrate transporter 2.5 | Potri.015G081300 | TTGCTATGGGGACTGCTTGT | ATGAGGCGATGGAAGTGAAG |
| Protein NRT1/ PTR FAMILY 6.4 | Potri.002G225500 | TGGAGGGTATCGTTATTGGC | GAGTAGGGAACTGTGGCA |
| Ammonium transporter 1 member 2 | Potri.002G255100 | TCTGACCTCCAGTCCTTAC | AATGGTCTGAAACCGCAAC |
| Inorganic phosphate transporter 1-11 | Potri.015G022800 | CTCGGTCGCCTTTATTACT | AGCCCAACCGAAGACTAAT |
| Probable potassium transporter 13 | Potri.005G095900 | CATCCCCCATCTATGTCTAC | GGAGTGTCGGCAGAGTAACG |
| Probable cadmium/zinc-transporting ATPase HMA1, chloroplastic (Precursor) | Potri.007G049000 | CCGATGTTCTACTGCTAAGG | GTAAAAGAACCGTCAACCAG |
| Calcium-transporting ATPase 12, plasma membrane-type | Potri.013G038400 | TACCAGAGCCGAAACGCATT | ATCTCGGTCTGGCTGTCAAG |
| Manganese-dependent ADP-ribose/CDP-alcohol diphosphatase | Potri.015G085200 | CTCATTTGGTGTAATCTCTG | ATTCCACTTTTTTACTGCCC |
| Sugar transporter ERD6-like 2 | Potri.005G037300 | GCTGGGACATTCTTCATCTT | CTGGCACCAACTTCCATACG |
| Sugar carrier protein C | Potri.T018200 | TTGGTATCTCGGGTGGTGTG | ATGTGAACAAGGTTAGCCCC |
| **Genes associated with disease and stress resistance** | | | |
| GDSL esterase/lipase At2g38180 (Precursor) | Potri.002G219700 | TGGGACTGGCTATGACAATG | ATCGCCTGGTTGGAAATGTGT |
| Laccase-14 (Precursor) | Potri.019G088500 | AAGAAGGAACTCTGTGGTGGC | TAAGGTGCGGGAAACGGATA |
| Putative disease resistance protein RGA3 | Potri.012G123200 | ACCGATGAACTACAACGACT | TCACCCACCAACTTGCTTAC |
| Pectinesterase 1 (Precursor) | Potri.001G162600 | CAGTAGAAATGAAAGTGGGT | GTAAACACCTGCCTTGATTC |
| Thaumatin-like protein 1 (Precursor) | Potri.001G221100 | GAACTCTAACGGCTGCTGGC | CAAACTTTCCCGAGGCATCT |
| Peroxidase C3 (Precursor) | Potri.001G013000 | ATAGAGAGCGAAAAGGAAGC | AAACAGTAGTAGGACAGGCA |
| Stress-related protein | Potri.014G131100 | CTAAACAGCGAACAGAGATG | ACGCTTGAAAAACAGACCACC |
| Wound-responsive protein GWIN3 (Precursor) | Potri.019G124500 | CCAAGTTTGTAGTGCTCTCC | CCTCATTACCGTTGACATCG |
| Mechanosensitive ion channel protein 2, chloroplastic (Precursor) | Potri.005G107000 | TAGGCTCCTCTACTCAAG | GTTTTCGTCTGAACCCAT |
| Pleiotropic drug resistance protein 3 | Potri.010G153600 | GTAGAGGAGGATGAGGGAGT | ATTGCTTGCTTCAAACAGTG |
| Chitinase 2 | Potri.005G059400 | AGTCTTTCTGGTTGGAGTTT | TAGGGAAGTTTTCGTAATCG |
| Heat shock cognate 70 kDa protein 2 | Potri.008G054800 | TAGAGCGTCTGATTGGTGAT | ATGGTAAGCCTCGTTGTAGT |
| Chitin-inducible gibberellin-responsive protein 1 | Potri.001G409500 | GTCCTTCTCGTAACTCTTCA | TCTGTGGCGTGCTACTATC |
| **Genes associated with cell structure** |  |  |  |
| Vegetative cell wall protein gp1 (Precursor) | Potri.002G252400 | GTGTTAGTGCTTGCTCTTG | GAACCCACCATCTAAAGGA |
| Cell number regulator 2 | Potri.008G132800 | GTATCCATCAAAGCAAGAC | AGAGAAGCAGTCACAAAGA |
| Extensin (Precursor) | Potri.002G243200 | CAGCCTTGGAACTTTTACGG | CACATTTTGTAGGGGCATT |
| 65-kDa microtubule-associated protein 3 | Potri.006G269800 | GGGTTGAGAAATGGTTGACT | GCACGCTTGAGAGTTAGATG |
| Wall-associated receptor kinase-like 8 (Precursor) | Potri.004G192700 | GCCAATAAGACAAGTGCGTA | ACCAGTTTTACCATCACCGT |
| Cell division control protein 48 homolog A | Potri.001G128700 | AGCAAAGTCAACAACGGAAC | GCCATTTTAGACCGAGTTTC |
| **Genes** **associated with hormone synthesis and metabolism** | | | |
| Auxilin-related protein 2 | Potri.002G217200 | TTTACTCAACCCACAAGCG | TCGTCAACAGGTAGAACAT |
| Auxin response factor 6 | Potri.002G055000 | TGATGTTGAGACAGACGAAG | ATAGTTAGACGGTTGTTTGC |
| Ethylene-responsive transcription factor ERF017 | Potri.006G218200 | CGTCATCTTCGTCAGTGTC | TTTCGGGCGTGTCATAGGA |
| ABSCISIC ACID-INSENSITIVE 5-like protein 1 | Potri.009G164500 | TAACGAAGCAATGTCCGAAT | CTTCTAATGTTATCTCACCC |
| Gibberellin 20 oxidase 2 | Potri.015G002800 | TCCTCTGGCGTCCATTACAC | CTCGCAGGCATCACCAACTT |
| **Genes associated with MYB transcription factor** | | | |
| Transcription factor MYB114 | Potri.017G125900 | ATGGACCGAAGAGGAGGATA | TCGCCCACTGAAAACTGTCC |
| Transcription repressor MYB5 | Potri.019G036300 | CAACAACTTCAACAAGGAGC | TACCACACCTGAGAAGTCCT |
| Transcription factor MYB86 | Potri.003G155700 | ATGGTGAGCTGTGATGATATGCAGG | CCTGCATATCATCACAGCTCACCAT |
| Transcription factor bHLH79 | Potri.012G072700 | CGGGGTCAAGGAATGAAAAC | TCTCTGCTAAACTGTGGCT |
| **Genes associated with senescence** | | | |
| Senescence-specific cysteine protease SAG39 | Potri.005G088600 | CAATGGATGGCTCAGTATG | GACACCGAGTTTGTAAGAT |
| Senescence-associated carboxylesterase 101 (Precursor) | Potri.001G290600 | GGTTACTACGACAGTTACA | TTTCTGGGGTTTTCGCTCA |
